# Malignant Degeneration to Leiomyosarcoma of the Anorectal Junction Invading the Prostate

**DOI:** 10.7759/cureus.19196

**Published:** 2021-11-01

**Authors:** Gabrielle Perrotti, Olivier Van Houtte, Amanda Ayers, Erica Lambert, Robert Lewis

**Affiliations:** 1 General Surgery, Jefferson Abington Hospital, Abington, USA; 2 Colorectal Surgery, St. Francis Hospital and Medical Center, Hartford, USA; 3 Urology, St. Francis Hospital and Medical Center, Hartford, USA

**Keywords:** leiomyosarcoma, other neoplasia, anorectal junction, anal canal, gleason prostate cancer

## Abstract

While colonic leiomyomas are common, leiomyosarcomas of the GI tract are rare. Increased mitotic rate, as well as lymph node involvement, portend a worse prognosis in leiomyosarcomas. They can arise anywhere along the GI tract, but anal canal occurrence is extremely rare.

We present the case of a 75-year-old male diagnosed eight years prior with leiomyoma of the anorectal junction. There was a recurrence after endoscopic resection. He was referred to colorectal surgery due to symptoms of bleeding, skin irritation, anal pruritus, and rectal pain. On exam, he had a palpable mass at the dentate line. Workup revealed a 3.5 cm mass at the anorectal junction with pathology showing a leiomyosarcoma with moderate atypia and a high mitotic rate. MRI revealed invasion into the prostate. Robotic pelvic exenteration, including cystoprostatectomy, abdominoperineal resection, and ileal conduit, was performed. Final pathology results showed a grade 2 leiomyosarcoma invading the prostate and skeletal muscle and incidentally found Gleason 3+4 prostate cancer, pT2.

A very small percentage of anorectal leiomyosarcoma cases were located in the anal canal. Surgery remains the best curative option, as chemotherapy and radiation data are limited. This rare tumor, which previously has not been documented to have degenerated from a benign rectal leiomyoma, was diagnosed as a result of close monitoring after previous local resections and cured by local resection and radiation.

## Introduction

Stromal neoplasms that arise in the GI tract are grouped into two major divisions. The first is GI stromal tumors (GISTs) which are common and predominantly arise in the stomach and proximal small intestine. The second group comprises several neoplasms and includes leiomyomas, leiomyosarcomas, desmoid tumors, schwannomas, and peripheral nerve sheath tumors [1].

Overall, leiomyomas of the GI tract are uncommon and leiomyosarcomas are quite rare. Although leiomyosarcomas can resemble GISTs, they show features of smooth muscle differentiation and generally are an aggressive tumor with cellular atypia, abundant mitosis, and include areas of necrosis [2]. Leiomyosarcomas, unlike GISTs, are negative for KIT, CD 34, and DOG-1. Further differentiation of leiomyosarcomas from leiomyomas is categorized by the presence of cellular atypia and high mitotic activity of greater than 50 per 50 high power fields [3]. They are commonly found to bulge intraluminally and are a polypoid mass with histologic appearances similar to smooth muscle cells [4]. Associated with TP 53 carriers and commonly arising in patients with a history of retinoblastoma, leiomyosarcomas can arise anywhere in the GI tract from the esophagus to the rectum. They appear to spread hematogenously to the lungs and locally to surrounding organs. The majority occur in the small bowel and the second most common anatomical location is the colon [2].

Colonic leiomyosarcomas are highly aggressive tumors irrespective of their tumor size or mitotic activity. On the other hand, rectal leiomyosarcomas are generally small and have prolonged patient survival with an overall more favorable prognosis. This is likely secondary to the polypoid nature of the tumors, which allows for easy palpation on rectal exams and sooner discovery by physicians [2]. Only four cases of anorectal leiomyosarcoma had been reported in the literature [3].

## Case presentation

We present the case of a 75-year-old male who was referred to the colorectal surgery clinic for evaluation after noting hematochezia, anal skin irritation, and rectal pain. Eight years prior, he had been diagnosed with leiomyoma of the rectum. At that time, he had undergone a biopsy of the lesion and local resection was performed several times. After each resection and biopsy, the pathology proved to be a benign leiomyoma. 

**Figure 1:**
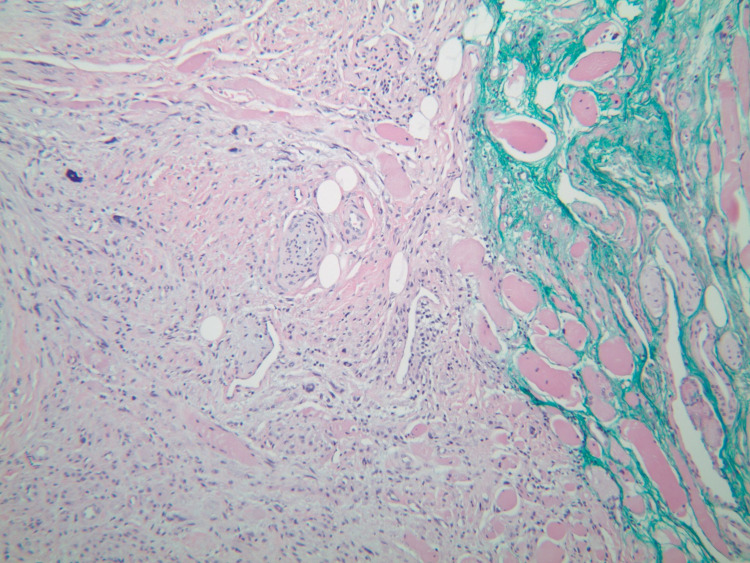
Leiomyosarcoma tumor seen invading skeletal muscle.

**Figure 2:**
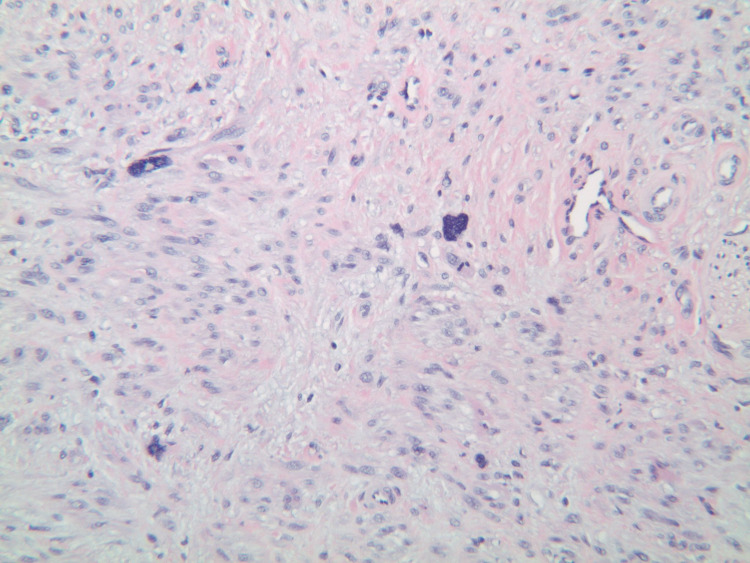
Patient's biopsy with H&E staining.

**Figure 3:**
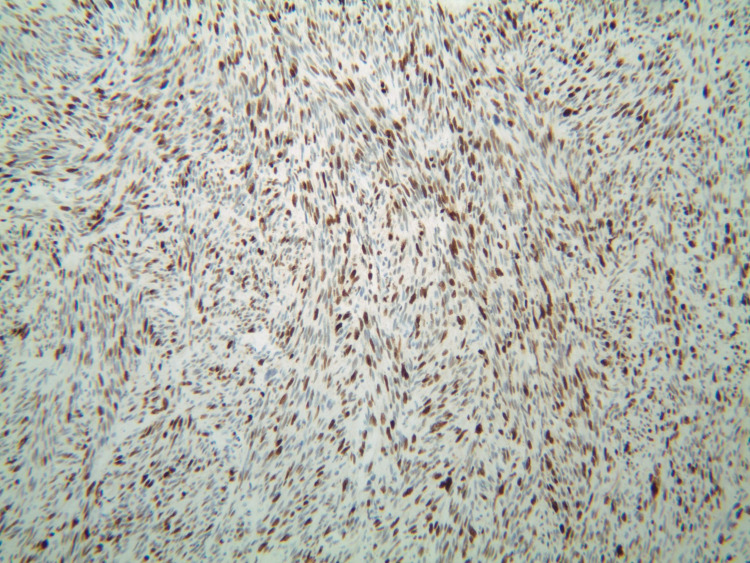
Patient's biopsy with Ki67 staining.

**Figure 4:**
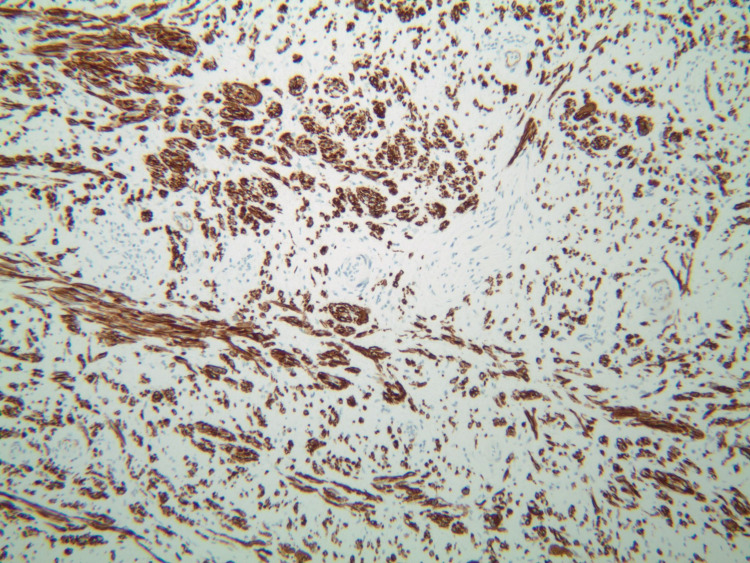
Patient's biopsy with desmin staining.

Upon his latest presentation, eight years after initial local resection, a physical exam revealed a palpable mass at the anorectal junction. Biopsy of the mass was now consistent with leiomyosarcoma, including moderate atypia and a high mitotic rate (40 per 10 high power field), as compared to his previous diagnosis of leiomyoma (Figures 1-4).

**Figure 5:**
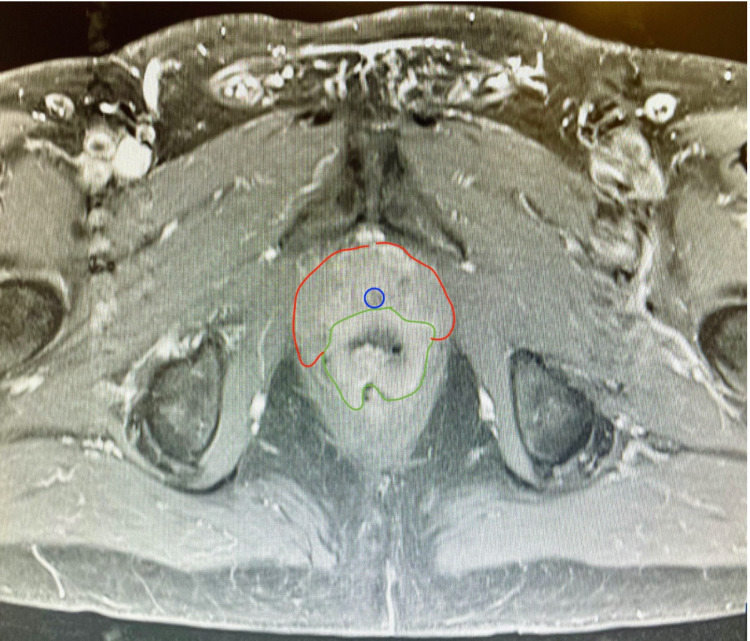
MRI of the pelvis showing the rectal leiomyosarcoma (green) as it invades anterior into the prostate (red) and nears the urethra (blue).

He underwent staging workup with an MRI of the pelvis (Figure 5). This proved the mass to be 3.5 cm in diameter, located at the anorectal junction, with extension into the prostate and possible involvement of the urethra. The decision was made for the patient to undergo surgery. The colorectal team, combined with urological surgical teams, performed a robotic pelvic exenteration, which included cystoprostatectomy, abdominoperineal resection, and ileal conduit creation.

The final pathology resulted in a grade 2 leiomyosarcoma that invaded the prostate and skeletal muscle. Zero of 26 lymph nodes were positive. Incidentally, he was found to have Gleason 3+4 prostate cancer that was staged as pT2. The postoperative course was uncomplicated and he was discharged home from the hospital.

As an outpatient, he completed adjuvant radiation therapy to prevent local recurrence of his leiomyosarcoma. Complete surgical resection cured his prostate cancer, but urology will continue to monitor him for recurrence with interval PSA levels. As for monitoring of his leiomyosarcoma, he will continue to undergo regularly scheduled physical exams and imaging to assess the recurrence or development of metastatic disease. He is currently free of disease 18 months after surgery based on his most recent MRI scan and physical exam.

## Discussion

Our case represents a rare disease process of anorectal leiomyosarcoma. Leiomyomas are more commonly found in uterine tissue, but can occur in any location with smooth muscle cells. Degeneration of leiomyomas has been described in uterine tissue, once again, but due to its rare nature, it is less well described in the anorectal region [5]. In this instance, the reason the histologic change was identified is due to the close monitoring the patient underwent after previous local resections.

According to a 2019 systematic review of the literature for the last 20 years, there were only a total of 51 cases of anal or rectal leiomyosarcoma reported. This is estimated to be less than 0.1% of all cases of anorectal malignancies. Of those 51 reported cases of anal and rectal leiomyosarcoma, only four cases, or 8%, were located solely in the anal canal [3]. This case serves to demonstrate the rare nature of the entity and its invasion of the prostate shows its aggressive behavior.

Surgical resection with negative margins is the best curative option. Local resection or endoscopic treatment for benign leiomyomas is reasonable, as was performed previously, but the leiomyosarcoma needs to be addressed with radical resection. The disease progresses by local spread or invasion and does not tend to travel to lymph nodes. In cases of R1 or R2 resection, re-excision is indicated since positive margins are a predictor of local recurrence. Local recurrence after wide local excision was cited as having a 30% recurrence rate compared to radical resection which had a 20% rate. Radical resection and extensive surgical procedures such as en bloc resection of pelvic exenteration were reserved for tumors invading adjacent organs, as in our patient. Interestingly, distant metastasis was found to have a higher incidence in patients who underwent radical resection [3].

Regardless of the treatment approach, metastasis of leiomyosarcomas were cited at an overall rate of 51.61%. Characteristics such as a mitotic rate greater than or equal to 10/10 high power field, high tumor grade, and/or a tumor size larger than 10 cm were associated with a higher risk of metastasis and worse survival. Lymphadenectomy is not an indicated procedure during resection as leiomyosarcomas do not generally metastasize to lymph nodes. However, if local regional lymph nodes are enlarged on preoperative imaging, then resection is indicated. Data on the use of chemotherapy and radiation is limited. Neoadjuvant radiation is thought to help with the R0 resection of the tumors compared to adjuvant radiation. However, patients with local invasion often undergo radiation therapy afterward, as seen in our patient, to prevent a recurrence. Additionally, those cases that received adjuvant chemotherapy had better rates of overall survival compared with those that did not. Although multiple trials have assessed different regimens, no regimen has yet to be proven to be the top choice for leiomyosarcomas of the abdomen. Doxorubicin-based regimens remain the standard first-line choice of chemotherapy regimen for metastatic or locally advanced soft tissue sarcomas [3].

## Conclusions

Leiomyomas are rare neoplasms of the GI tract and particularly of the anal canal. Their malignant counterpart, leiomyosarcomas, behaves aggressively and can spread locally to other organs. They are best worked up with MRI or endorectal ultrasound to determine the depth and possible invasion. They are best treated with radical surgery to resect any local disease. Discussion with a multidisciplinary tumor board should address the possibility of radiation and chemotherapy as an adjunct to the surgical plan. Overall survival is poor, but early detection and aggressive therapy lead to the best outcomes.
